# Salicylic acid inducing the expression of maize anti-insect gene *SPI*: a potential control strategy for *Ostrinia furnacalis*

**DOI:** 10.1186/s12870-024-04855-6

**Published:** 2024-02-29

**Authors:** Yuanlong Chen, Siyuan Yang, Wei Zeng, Xu Zheng, Pan Wang, Haiyan Fu, Fengshan Yang

**Affiliations:** 1 Engineering Research Center of Agricultural Microbiology Technology, Ministry of Education & Heilongjiang Provincial Key Laboratory of Ecological Restoration and Resource Utilization for Cold Region & Key Laboratory of Molecular Biology, College of Heilongjiang Province & School of Life Sciences, Heilongjiang University, Harbin, 150080 China; 2 School of economies and management, Beijing University of chemical technology, Beijing, 100029 China

**Keywords:** Induces defense, Serine protease inhibitors, Resistance to insects, Transcriptome analysis, Fusion protein

## Abstract

**Background:**

Due to being rooted in the ground, maize (*Zea mays* L.) is unable to actively escape the attacks of herbivorous insects such as the Asian corn borer (*Ostrinia furnacalis*). In contrast to the passive damage, plants have evolved defense mechanisms to protect themselves from herbivores. Salicylic acid, a widely present endogenous hormone in plants, has been found to play an important role in inducing plant resistance to insects. In this study, we screened and identified the insect resistance gene *SPI*, which is simultaneously induced by SA and *O. furnacalis* feeding, through preliminary transcriptome data analysis. The functional validation of *SPI* was carried out using bioinformatics, RT-qPCR, and heterologous expression protein feeding assays.

**Results:**

Both SA and *O. furnacalis* treatment increased the expression abundance of SA-synthesis pathway genes and *SPI* in three maize strains, and the upregulation of *SPI* was observed strongly at 6 hours post-treatment. The expression of *SPI* showed a temporal relationship with SA pathway genes, indicating that *SPI* is a downstream defense gene regulated by SA. Protein feeding assays using two different expression vectors demonstrated that the variation in SPI protein activity among different strains is mainly due to protein modifications.

**Conclusions:**

Our research results indicate that *SPI*, as a downstream defense gene regulated by SA, is induced by SA and participates in maize's insect resistance. The differential expression levels of *SPI* gene and protein modifications among different maize strains are one of the reasons for the variation in insect resistance. This study provides new insights into ecological pest control in maize and valuable insights into plant responses to SA-induced insect resistance.

**Supplementary Information:**

The online version contains supplementary material available at 10.1186/s12870-024-04855-6.

## Background

As a plant firmly rooted in the ground, maize (*Zea mays* L.) lacks the ability to actively avoid or escape attacks from herbivorous insects such as the Asian corn borer (*Ostrinia furnacalis*), which has been a significant threat to maize production in China in recent years [1–3]. In contrast to passive damage, plants have evolved unique defense mechanisms through a long process of evolution to protect themselves from herbivores [4–6].

Salicylic acid (SA) is a naturally occurring endogenous hormone in plants and has been found to play important roles in stress resistance, disease resistance, and insect defense [7–9]. When insects feed on plants, the SA pathway is activated, leading to the transcriptional expression of downstream defense genes. This results in the production of toxins or defense proteins in plants to resist insect damage, thereby enhancing their own insect resistance and reducing insect damage [9–11]. Recent studies have shown that the feeding process of *Myzus persicae* induces the expression of SA-related genes in *Arabidopsis* [12]. The *Diuraphis noxia Mordvilko* induces the accumulation, conversion, and transport of SA during its feeding on wheat [10, 13]. The resistance of tomato to potato aphids mediated by the *Mi-1* gene depends on SA [14]. Studies on the feeding behavior of *Helicoverpa armigera Hubner* larvae have shown that the SA pathway also plays an inhibitory role in the feeding of chewing insects [15, 16].

Serine protease inhibitors (SPIs) are widely distributed proteins in plants [17, 18] and constitute the largest and most diverse family of protease inhibitors [19–21]. SPIs play a crucial role in protecting plants against insect and pathogen attacks [22, 23]. They can irreversibly alter the structure of serine and cysteine proteases, rendering them inactive [24–26]. It has been found that the digestive enzymes of lepidopteran and coleopteran insects are predominantly serine proteases. When insects feed on plants, SPIs accumulate in their bodies, inhibiting the activity of insect digestive enzymes and reducing nutrient absorption and utilization, thereby exerting an insecticidal effect.

**Fig. 1:**
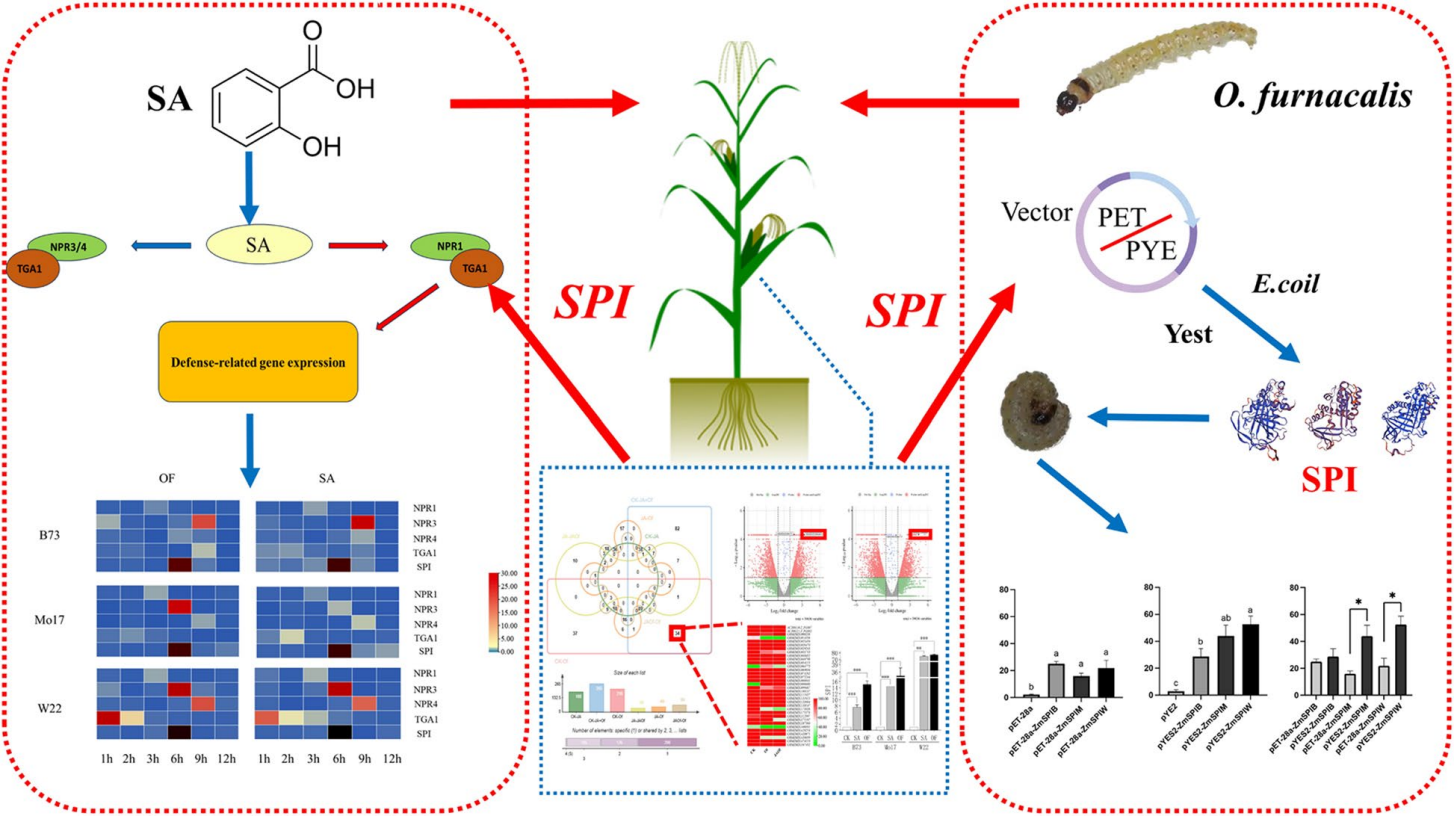
Research focus and technical route

In this study, we screened and identified *SPI* genes that are simultaneously induced by SA and *O. furnacalis* feeding by analyzing the transcriptional changes of defense genes in maize after *O. furnacalis* feeding using previous transcriptomic data. We further investigated the relationship between SA conduction response marker genes and SPI through temporal expression analysis. We successfully identified and functionally validated the *SPI* genes, and clarified the relationship between induced resistance in inbred lines and the expression of *SPI* and the downstream signaling pathway of SA (Fig. 1). This provides new insights into the ecological control of corn pests and valuable insights into the plant's response to SA-induced insect resistance.

Through the transcriptome analysis of JA-treated maize, potential genes induced by SA, *SPI*, were identified and the differential expression of *SPI* between inbred lines was analyzed using bioinformatics. Real-time fluorescence quantitative analysis was conducted to investigate the interaction between key genes in the SA pathway and *SPI* genes in the context of *O. furnacalis* feeding and SA treatment, confirming the regulation of *SPI* by the SA pathway. Finally, the differences in anti-feedant activity of SPI protein in three maize inbred lines were evaluated through exogenous expression of SPI in prokaryotic and eukaryotic systems.

## Result

### SA treatment conditions and cultivar selection

**Fig. 2:**
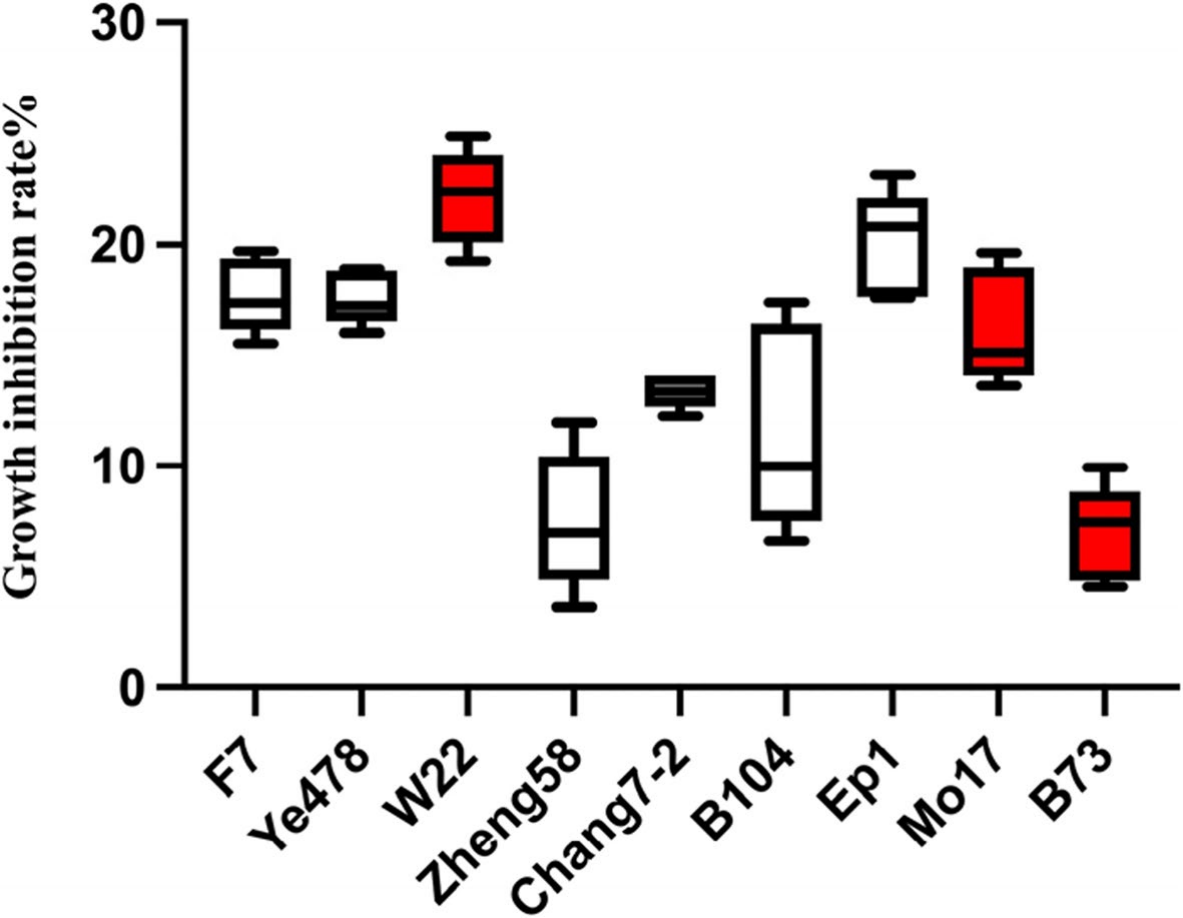
Effects of SA - induced maize inbred lines on growth inhibition rate of *O. furnacalis* larvae

Different concentrations and durations of SA induction treatment on maize leaves have varying effects on the developmental period and growth of *O. furnacalis* (Table 1). Significant differences in insect resistance were observed also among different maize varieties under SA induction (Fig. 2). The growth inhibition rate was determined after feeding *O. furnacalis* with maize leaves induced by different concentrations of SA, and the optimal concentration was found to be 0.5 mM, with an optimal induction time of 6 hours. Feeding on this time and concentration conditions SA-treated maize leaves resulted in growth inhibition of the *O. furnacalis*, with growth inhibition rates ranging from 6.95% to 22.14%. In this study, we selected the inbred lines W22 (highest), Mo17 (median), and B73 (lowest), which showed different levels of growth inhibition after induction, for further experiments.

**Table 1:** Effects of feeding SA treatment maize leaves on the growth and development of *O. furnacalis*

	SA concentration (mM)			
		Time		
		3h	6h	9h
Larval instar (d)	0	16.50 ± 0.74Ca	16.40 ± 1.16Da	16.10 ± 1.46Ca
	0.1	16.00 ± 1.10Ca	17.50 ± 1.62Ca	17.70 ± 0.92Aa
	0.5	19.60 ± 1.06Bb	21.90 ± 0.25Aa	19.20 ± 1.47Bb
	1	20.00 ± 2.25Aa	21.00 ± 1.05Ba	19.60 ± 1.65Ba
Pupa (d)	0	6.52 ± 0.98Ca	6.13 ± 0.14Da	5.37 ± 0.28Ca
	0.1	7.18 ± 0.61Ba	7.12 ± 0.34Ca	6.69 ± 0.53Aa
	0.5	8.00 ± 0.44Aa	8.49 ± 0.43Aa	7.56 ± 0.46Ab
	1	7.92 ± 0.49Ba	7.69 ± 0.73Ba	6.38 ± 0.30Bb
Growth inhibition rate (%)	0.1	5.19 ± 2.30Bb	16.71 ± 0.94Ba	15.70 ± 3.17Ba
	0.5	24.15 ± 0.52Ab	28.39 ± 3.82Aa	27.22 ± 1.97Aa
	1	23.24 ± 2.94Ab	27.12 ± 1.89Aab	31.15 ± 0.45Aa

^*^Capital letters are ANOVA results of different induction concentrations at the same induction time; Lowercase letters indicate ANOVA results for different induction times at the same induction concentration. *P value* <0.05 were considered to be significant

### The *O. furnacalis* feeding activated the SA synthetic pathway in maize

**Fig. 3:**
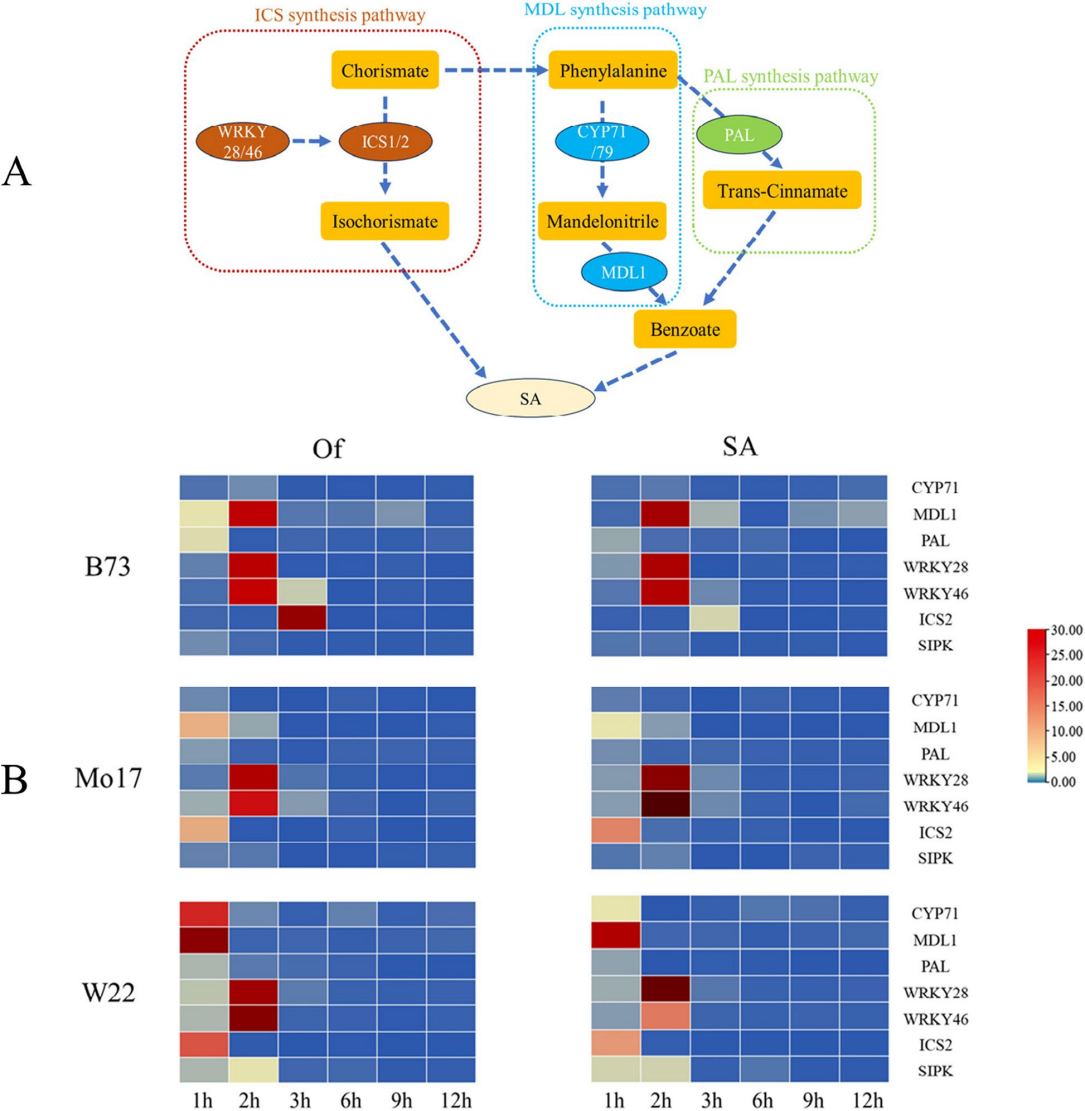
Relationship of SA- synthesis pathway key genes (**A**), Time series analysis of transcription abundance of SA- synthesis pathway key genes (**B**)

When the three inbred lines were stimulated by *O. furnacalis* feeding, the transcription levels of the upstream regulatory factor *SIPK* in the SA synthesis pathway increased. In all three inbred lines, the transcription levels of *SIPK* significantly increased 1-2 hours after feeding. B73 and W22 showed decreased transcription levels 2 hours after feeding, and returned to the resting state after 3 hours of feeding. Mo17 showed significantly lower transcription levels 3-6 hours after feeding compared to the control group, and returned to the resting state after 9 hours of feeding (Fig. 3).

Activation of SIPK can regulate the activation of the SA synthesis pathway. The known SA synthesis pathways are the phenylalanine synthesis (PAL) synthesis pathway and the isochorismate synthesis (ICS) synthesis pathway (Table 2). The transcription levels of key genes in the SA synthesis pathway were determined among different inbred lines. In the different inbred lines, *PAL* showed a significant increase in transcription levels 1 hour after feeding, indicating activation of the phenylalanine synthesis pathway. The transcription levels of B73 and Mo17 returned to the resting state 2 hours after feeding, while W22 maintained relatively high levels of transcription between 2-3 hours after feeding, at 3.58 and 2.22 times higher, respectively. *WRKY28*, *WRKY46*, and *ICS1* are key genes in the isochorismate synthesis pathway. In B73, the transcription levels of *WRKY28* significantly increased 1-2 hours after feeding, *WRKY46* significantly increased 2-3 hours after feeding, and *ICS1* significantly increased 3 hours after feeding, indicating activation of the isochorismate synthesis pathway. Mo17 and W22 showed increased transcription levels of *WRKY28* and *WRKY46* 1 hour after feeding, along with a significant increase in *ICS1* transcription levels, indicating activation of the isochorismate synthesis pathway.

**Table 2:** SA synthetic pathway key genes

		MaizeGebB ID		
SA Pathway	Gene	B73	Mo17	W22
MDL synthesis pathway	*CYP71*	Zm00001eb008985	Zm00014a012266	Zm00004b025195
	*MDL1*	Zm00001eb036701	Zm00014a029117	Zm00004b029447
PAL synthesis pathway	*PAL*	Zm00001eb247610	Zm00014a010744	Zm00004b014426
ICS synthesis pathway	*WRKY28*	Zm00001eb388620	Zm00014a038524	Zm00004b033076
	*WRKY46*	Zm00001eb098330	Zm00014a018738	Zm00004b008900
	*ICS2*	Zm00001eb310840	Zm00014a014675	Zm00004b035774
Transcription factor	*SIPK*	Zm00001eb393610	Zm00014a037968	Zm00004b033522

Malonyldialdehyde (MDL) synthesis pathway is a newly discovered SA synthesis pathway, and the key genes *CYP71* and *MDL1* showed increased transcription levels within 1 hour after *O. furnacalis* feeding, initiating SA synthesis. There were differences in the duration of high transcription levels of these two genes among the different inbred lines. *CYP71* returned to the resting state 3 hours after feeding in both B73 and W22, while Mo17 returned 2 hours after feeding. *MDL1* in the three inbred lines returned to the resting state after 12 hours, 3 hours, and 2 hours of feeding, respectively.

Additionally, comparing the feeding of *O. furnacalis* with exogenous SA treatment of maize leaves revealed that the trends in SA synthesis gene changes were similar under both treatments. This demonstrates that *O. furnacalis* feeding promotes the expression of the SA synthesis pathway and suggests that the SA pathway may be involved in maize defense against *O. furnacalis* (Fig. 3).

### Screening and identification of potential SA-induced maize insect resistance genes

**Fig. 4:**
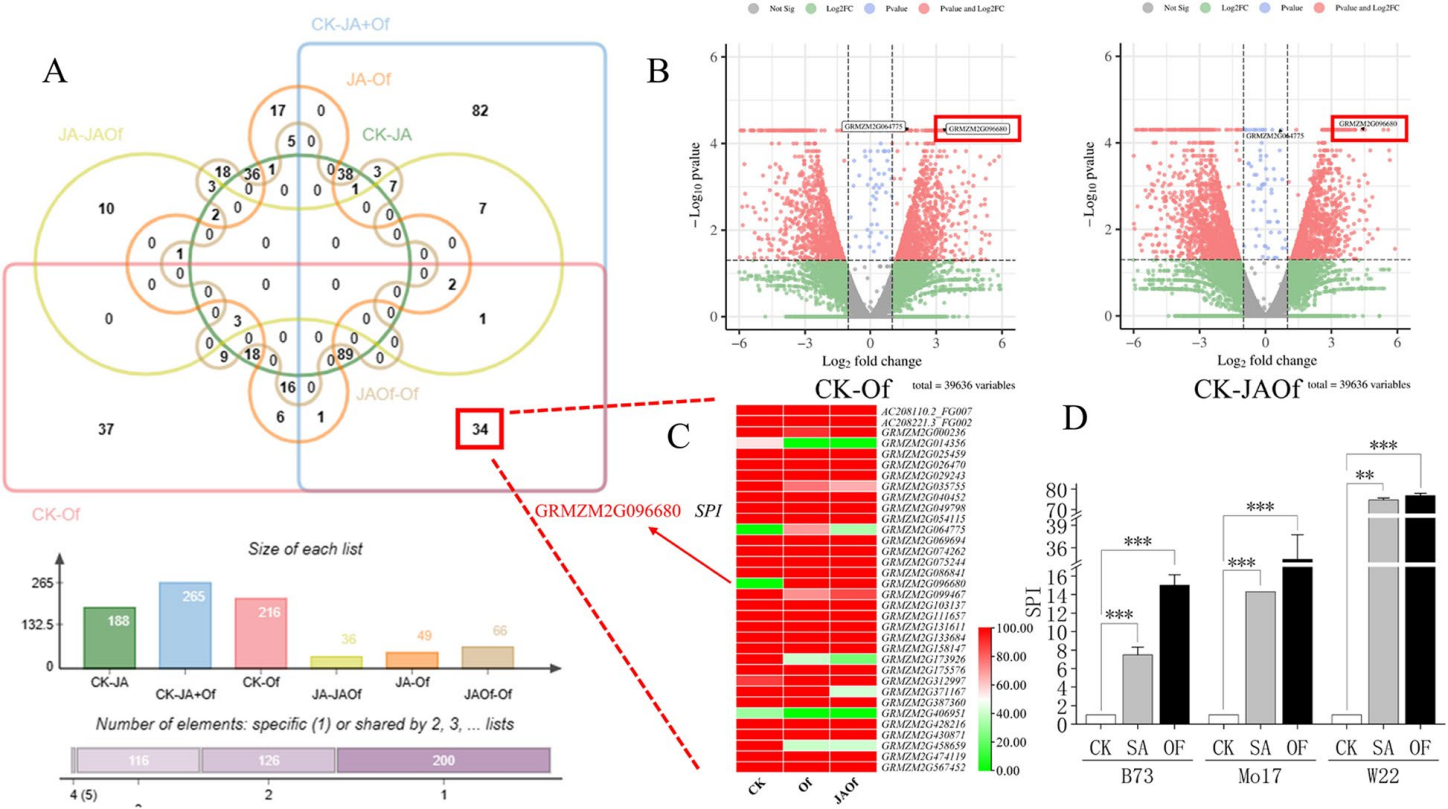
Transcriptome data used to identify *SPI* by vnn diagram of differentially expressed genes (**A**), volcano plot of different treatment (**B**), heat map of co-expressed genes between CK – Of and CK – JAOf (**C**), and transcript abundance of *SPI* after SA and Of treatments in three maize cultivars (**D**). [Letters on the error bars indicate significant differences analyzed using the one-way ANOVA followed by Duncan's text. (* P < 0.05, ** P < 0.01, *** P < 0.001, *∗∗∗∗ P* < 0.0001)]

Based on previous transcriptome data analysis of insect feeding on commercial maize variety Longdan 46, it was found that there were 216 significantly upregulated genes in maize tissues after *O. furnacalis* feeding, while there were 265 significantly upregulated genes in maize tissues after co-treatment with combined treatment of methyl jasmonate and *O. furnacalis*. Nevertheless, among 34 genes that were upregulated only in the treatment with *O. furnacalis* feeding or methyl jasmonate and *O. furnacalis* (Fig. 4A), we found these two genes, GRMZM2G096680 (*SPI*) and GRMZM2G064775 (*ZIM*), that were strongly induced by *O. furnacalis* infestation alone or in combination with MeJA treatment, but not by MeJA treatment alone. (Fig. 4C). The volcano plot showed that ZIM was only significant in terms of p-value after the JAOf treatment (Fig. 4B). We speculated that this gene might not be regulated by the JA pathway. Given that JA and SA pathways are known to antagonize each other, we boldly hypothesized whether this *SPI* gene might be upregulated by the SA pathway. Therefore, we selected *SPI* as the potential gene for further experiments.

The expression of *SPI* in the three maize inbred lines was detected using qRT-PCR after *O. furnacalis* feeding and SA induction (Fig. 4D). The relative expression of *SPI* was highest in W22 and lowest in B73 after insect feeding, which is consistent with the pattern of maize insect resistance (Fig. 2).

### Differences in the *SPI* sequence and protein structure among different maize inbred lines

To investigate the differences in the SPIs among different maize varieties, the gene and protein sequences of SPI were compared among different maize inbred lines. The alignment of *SPI* sequences from the three inbred lines is shown in Fig. S1B, with open reading frames (ORFs) ranging from 1083bp to 1194bp in length. The sequence alignment consistency among the three maize inbred lines was only 76.16%.

Significant differences were observed in the conserved domains and exon structures of the SPI gene among different maize inbred lines, while the structural elements and promoter sequences showed minor differences. Three motifs were identified in the maize *SPI* analysis (Fig. S1Aa), with all three varieties containing 2 copies of Motif 2, 1 copy of Motif 1, and 1 copy of Motif 3. Three conserved domains were identified in the maize *SPI* analysis (Fig. S1Ab), with all varieties containing the SerpinP domain and the PHA02948 domain, while W22 also possessed the SPIP_plants domain. Analysis of the *SPI* promoter sequences revealed that the *SPI* could be induced by various hormones, including salicylic acid, methyl jasmonate, and abscisic acid (Fig. S1Ac). Analysis of introns and exons revealed that the *SPI* in maize contains 2 to 3 exons (Fig. S1Ad), indicating a simple gene structure. B73 contains 3 exons, while Mo17 and W22 contain 2 exons.

The sequences of the SPI protein were compared and analyzed for their physicochemical properties. The alignment of SPI protein sequences among the three maize inbred lines is shown in Fig. S1E, with amino acid lengths ranging from 360 to 397, and the protein sequence alignment consistency was 54.13%. The analysis of physicochemical properties revealed that the three SPI proteins had similar molecular weights, but significant differences in isoelectric point (pI), hydrophilicity, and instability index (Table S1). The protein molecular weights ranged from 38.81 to 42.15 kDa, pI ranged from 5.52 to 10.01, hydrophilicity (GRAVY) ranged from -0.210 to 0.076, and instability index ranged from 35.84 to 46.52. The SPI proteins of B73 and W22 were stable hydrophilic proteins, while the SPI protein of Mo17 was an unstable hydrophobic protein. The probability of signal peptides in the three SPI proteins ranged from 0 to 0.15%, indicating the absence of signal peptides in the proteins.

**Fig. 5:**
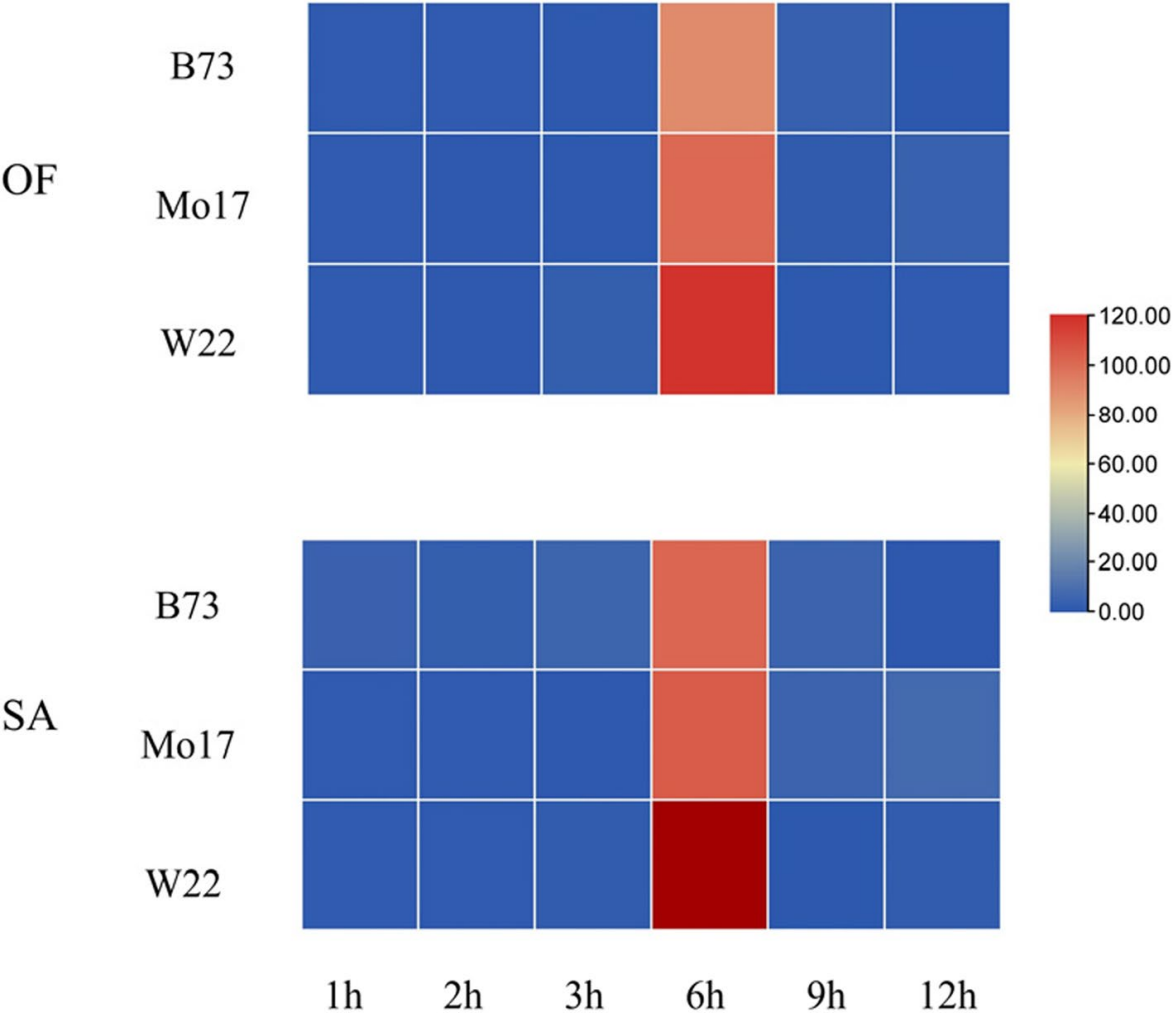
Time series analysis of transcription abundance of *SPI*

**Fig. 6:**
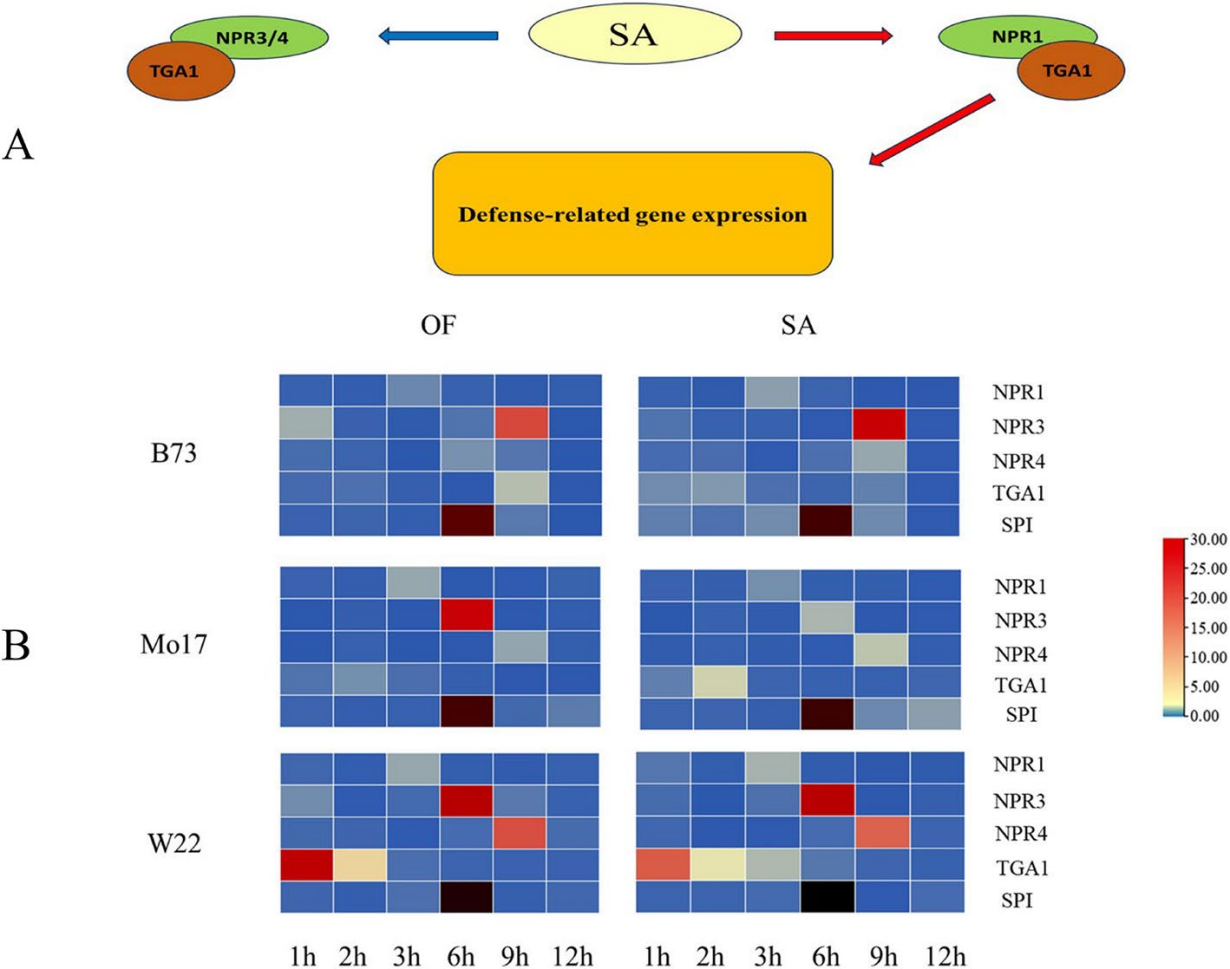
Relationship of SA-responsive marker genes (**A**), Time series analysis of transcription abundance of SA conduction response marker genes and *SPI* (**B**)

**Fig. 7:**
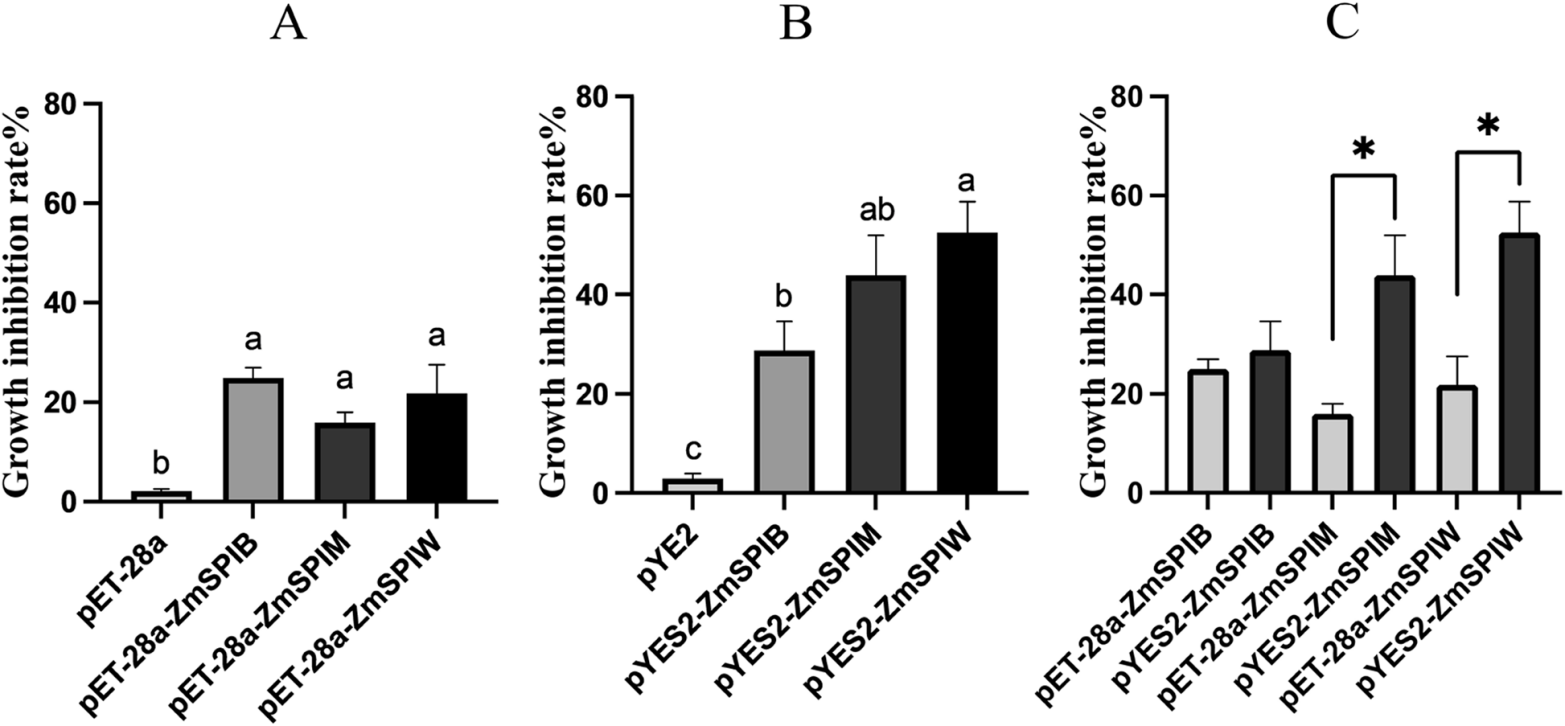
*O. furnacalis* larvae growth inhibition by feeding of lysate proteins expressed PET plasmids versus and three prokaryotic vectors (**A**), by feeding of lysate proteins expressed PYE plasmids versus and three eukaryotic vectors (**B**), differential analysis of proteins expressed by the two expression vectors (**C**)

Secondary structure prediction was performed for the three SPI proteins, as shown in Figs. 3, 4, 5, 6 and 7. The SPI proteins were composed of helical and coil structures, and the tertiary structure prediction was consistent with the secondary structure prediction results (Fig. S1C, D). There were significant differences in the tertiary structure of SPI proteins among the three inbred lines. The SPI protein of B73 had 31% α-helices, 32% β-extensions, and 4% TM helices. The SPI protein of Mo17 had 36% α-helices and 23% β-extensions. The SPI protein of W22 had 33% α-helices, 31% β-extensions, and 4% TM helices. According to the predicted transmembrane helix structure, both B73 and W22 proteins had transmembrane structures, with the C-terminus located outside the cell membrane and the N-terminus located inside the cell membrane.

### Expression of *SPI* in different inbred lines

After treatment with *O. furnacalis* feeding and SA treatment, the expression levels of SPI were detected by RT-qPCR, as shown in Fig. 5. The *SPI* in the three maize inbred lines were strongly upregulated at 6 hours after treatment. The heatmap and line graph showed that the expression levels of *SPI* in W22 were higher than those in Mo17 and B73, especially in the SA treatment group and the group treated with *O. furnacalis* feeding. The above results of real-time fluorescence quantification were consistent with the bioassay results.

### Temporal expression differences of marker genes in response to SA pathway in different inbred lines of maize

In order to further investigate the effects of *O. furnacalis* feeding and exogenous SA induction on the expression of key genes in the SA transduction pathway, three SA signaling genes from three maizes varieties were selected as response marker genes (Table 3). The relative expression levels of these three genes and *SPI* were measured at different time points under insect feeding and SA induction conditions. Among them, *NPR1* is a detection gene for initiating downstream defense genes in response to SA, while *NPR3/4* are termination detection genes for downstream defense genes (Fig. 6A). Real-time fluorescence quantification results showed that after *O. furnacalis* treatment and SA treatment, *NPR1* was upregulated at 3 hours in all three inbred lines of maize (Fig. 6B). The upregulation of *NPR3* in the B73 inbred line occurred at 1 hour and 9 hours after OF and SA treatments, with high expression at 9 hours. The upregulation of *NPR3* in the Mo17 line only occurred at 6 hours, while in the W22 line, it was upregulated at 1 hour and 6 hours, especially at 6 hours. Both Mo17 and W22 showed upregulation of *NBPR4* at 9 hours, with higher upregulation in W22 compared to Mo17. The *NBPR4* in the B73 line showed expression at 6 hours and 9 hours. As for *TGA1*, B73, Mo17, and W22 all showed upregulation at 1 hour and 2 hours after treatment, and B73 also showed upregulation at 9 hours, which was unique among the three lines. Overall, although there were slight differences in the expression levels of the four response marker genes between *O. furnacalis* treatment and SA treatment, the temporal upregulation patterns of the response marker genes were similar for both treatments. These results indicate that both *O. furnacalis* treatment and SA treatment can have similar effects on the SA signaling pathway, and exogenous SA treatment can enhance the defense response of maize similar to *O. furnacalis* feeding.

**Table 3:** SA-responsive marker genes

	MaizeGebB ID		
Gene	B73	Mo17	W22
*NPR1*	Zm00001eb370160	Zm00014a035285	Zm00004b028169
*NPR3*	Zm00001eb065420	Zm00014a014135	Zm00004b005853
*NPR4*	Zm00001eb116050	Zm00014a043478	Zm00004b010582
*TGA1*	Zm00001eb175150	Zm00004b021095	Zm00014a018212

### *SPI* vector expression and determination of growth inhibition rate

The *SPI*, *ZmSerpin-B*, *ZmSerpin-M*, and *ZmSerpin-W*, from three different inbred lines of maize were cloned, and the cloning results were confirmed by agarose gel electrophoresis. The PCR products of the *SPI* from all three varieties showed clear bands at around 1000 bp (Fig. S2), indicating successful cloning of the genes. The three genes were then separately ligated into the pET-28a and pYES2 vectors, and the ligated products were verified by double enzyme digestion (Fig. S3). The six recombinant vectors were transformed into *Escherichia coli* DH5α competent cells. After liquid culture of the positive clones, bacterial PCR was performed (Fig. S4). Plasmid extraction was carried out for the six recombinant plasmids transformed into DH5α, and the extracted pET-28a and pYES2 plasmids were separately transformed into *E. coli* BL21 and yeast competent cells. After transformation, colony PCR was performed to identify the positive clones (Fig. S5). *E. coli* BL21 and yeast cells containing the target gene were induced and then lysed for SDS-PAGE electrophoresis analysis (Fig. S6). The results showed that there was a clear band between the 34 KDa and 49 KDa bands of the protein marker in the lysates of both *E. coli* and yeast, with a size of approximately 42 KDa, concentration was approximately 0.2mg/ml, which matched the predicted size. This indicates that all vectors were successfully induced.

The SPI protein expressed in vitro was fed to second instar larvae of the *O. furnacalis*, and the relative inhibition rates were measured after 5 days (Fig. 7). The addition of the pET-28a and pYES2 empty vectors resulted in growth inhibition rates of 2.18% and 2.95%, respectively, which were not significantly different from the control group.

The SPI protein significantly affected the growth of *O. furnacalis* larvae, and both prokaryotic and eukaryotic expression systems showed significant growth inhibition effects on *O. furnacalis* larvae. When the *O. furnacalis* larvae fed on ZmSerpin-B, ZmSerpin-M, and ZmSerpin-W proteins expressed by the pET-28a vector, their growth and development were significantly inhibited, with growth inhibition rates of 24.95%, 15.95%, and 21.84%, respectively, and no significant difference was observed among the three proteins.

When the *O. furnacalis* larvae fed on the SPI protein expressed by the pYES2 vector, the growth inhibition rates for different inbred lines were 28.79%, 43.93%, and 52.57%, respectively, with W22 being the strongest, followed by Mo17, and B73 being the weakest. Significant differences were observed between W22 and B73. The activity of the protein expressed by the eukaryotic vector was higher than that of the prokaryotic vector, indicating that protein modification affects the activity of the SPI protein. The difference in insect resistance activity among the three SPI proteins is related to protein modification.

## Discussion

### SA synthesis induced by *O. furnacalis* feeding

Guo *et al*. found that after treating maize with *O. furnacalis*, a series of defense pathways, including phenylalanine, were involved in the defense response of mazie to the *O. furnacalis*[27]. This may lead to changes in the transcription abundance of the SA pathway. Although the content of SA in the *O. furnacalis* feeded mazie did not change significantly within 12 hours, there was still an upward trend in 2-4 hours. This is consistent with our research results. When we conducted real-time fluorescence quantitative detection of three key genes of three SA synthesis pathways in three maize inbred lines, we found that almost all SA synthesis pathway genes were up-regulated and then basically disappeared after 3 hours, with a peak in up-regulation of SA synthesis genes at 2 hours. This indicates that feeding by the *O. furnacalis* induces SA synthesis in plants within 4 hours. From 6 to 12 hours, downstream termination signals are activated, inhibiting the expression of upstream SA synthesis pathway genes. This is similar to the results of Guo *et al*., as the SA content gradually decreases from 4 to 12 hours.

Many studies on the induction of plant defense pathways by chewing insects have pointed out the role of the JA pathway in mediating the herbivore-induced response. Recent research has confirmed that SA also participates in plant defense against chewing insects, such as the activation of the SA pathway in tomatoes in response to feeding by cotton bollworm larvae. Salicylic acid-induced protein kinase (*SIPK*) is rapidly activated in plants when they are stimulated by external factors, which is consistent with our temporal expression results (Fig. 3B) [28]. Related studies have shown that tobacco rapidly activates *SIPK* when attacked by tobacco budworm, and SIPKs in turn activate other *MAPKs* and transcription factors [28–30]. When the three inbred lines are stimulated by *O. furnacalis*, the transcription levels of upstream regulatory factors in the SA synthesis pathway, including *SIPK*, increase, initiating the regulation of the SA synthesis pathway. Bernal *et al*. [31, 32] discovered a third pathway for the biosynthesis of salicylic acid from mandelonitrile (MD) in peach trees. We also identified the *MDL* gene in maize and studied its expression at different times in maize. The results show that under *O. furnacalis* feeding treatment, MDL pathway-related genes are expressed simultaneously with PAL pathway and ICS pathway genes, indicating the existence of a third pathway for mandelonitrile biosynthesis of SA in maize. This pathway is simultaneously activated with the other two pathways under external stimuli, but the specific pathway of MDL is not yet clear and requires further verification.

Under exogenous SA induction, the expression levels of key genes in the SA synthesis pathway do not exhibit negative feedback regulation, but instead increase in transcription levels similar to those induced by *O. furnacalis* feeding. Lydia and Sara's research found that SA synthesis genes *ICS1* and *PAL* can respond to exogenous SA induction, leading to an increase in transcription levels [33, 34].

Analysis of the temporal expression of the SA pathway among inbred lines revealed a close association between SA-induced insect resistance and the speed of SA pathway activation. Upon feeding by *O. furnacalis* and SA induction, the PAL pathway genes in all three inbred lines were transcribed at 1 hour of treatment. The MDL and ICS synthesis pathways were activated at 1 hour in the W22 and Mo17 varieties, while transcription was slower in the B73 variety, with the two synthesis pathways being activated at 2 hours and 3 hours, respectively. The PAL synthesis pathway plays a minor role in adjusting the SA content in plants; therefore, regardless of *O. furnacalis* feeding or SA induction, the transcription abundance of *PAL* genes did not show differences in timing among the different inbred lines.

As the induced insect resistance increased among the inbred lines, the transcription factor *TGA1*'s transcription initiation speed accelerated after *O. furnacalis* feeding, with the transcription levels increasing at 1 hour, 2 hours, and 9 hours for W22, Mo17, and B73, respectively. Additionally, there were differences in the transcription levels and the timing of transcription level increases of the insect-resistant feeding gene *SPI* among different inbred lines. In B73 and Mo17, SPI transcription levels significantly increased at 6 hours after feeding, while in W22, the transcription levels increased at 3 hours after feeding. *SPI* reached its maximum transcription levels at 6 hours after feeding in all three inbred lines, with levels being 22.55-fold, 25.20-fold, and 29.63-fold, respectively.

Under SA induction, *SPI* transcription levels significantly increased at 6 hours after induction in B73 and Mo17, while in W22, SPI gene transcription levels increased at 1 hour after induction. Therefore, the differences in the SA synthesis pathway among different inbred lines may be one of the important influencing factors for the differences in insect resistance in maize varieties.

### *SPI* exhibits insecticidal activity when induced by SA

This experiment is based on the previous analysis of transcriptomic data from maize subjected to insect feeding and SA induction in the laboratory. Through this analysis, we identified a potential defense gene, *SPI*, which showed significant upregulation in expression levels after both insect feeding and SA induction. This indicates that the *SPI* can be induced by SA and plays a role in maize's defense against insects. Further bioinformatics analysis of the *SPI* promoter revealed the presence of SA-responsive elements. To validate the function of the *SPI*, we constructed an exogenous expression system for maize *SPI* and conducted protein-based insect resistance assays. The results demonstrated that the SPI fusion protein from different maize inbred lines effectively inhibited the growth and development of the *O. furnacalis*. Insect feeding on proteins expressed by the pET-28a and pYES2 vectors significantly suppressed the growth and development of the insects. Previous studies have shown the positive impact of plant serine protease inhibitors on protecting crops from insect damage, and transgenic plants expressing serine protease inhibitors have exhibited resistance against pests such as the *Spodoptera littoralis* [22, 23, 35]. The serine protease inhibitor synthesized by *SPI* can inhibit insect growth and development by disrupting the function of digestive enzymes [36]. Additionally, Fernando *et al*. demonstrated significant growth inhibition of eight insect species, including the *O. furnacalis*, by *SPI* [37].

### The expression of the *SPI* is closely related to the SA pathway

At the current stage of research, most studies on SPI have focused on identifying its protein function and gene family. However, there is insufficient research on the regulation of SPI. In our study, we observed significant upregulation of *SPI* after SA induction in maize. To further investigate the relationship between *SPI* expression and SA induction, we analyzed the *SPI* promoter sequence. The results showed that the *SPI* promoter sequence contains regulatory sites for various hormones, including salicylic acid, methyl jasmonate, and abscisic acid, as well as binding sites for responding to exogenous stimuli (Fig. 4). This suggests that the SA pathway may regulate the transcription and expression of *SPI*. The promoter region is an important regulatory region for gene expression, and analyzing this region can infer the potential regulatory roles of specific sites in the transcription process.

Temporal expression data is widely used to study different dynamic biological processes and investigate gene regulatory networks based on the differential expression patterns of transcription factors and genes over time [38, 39]. In our study, we measured the expression of key genes in the SA pathway and *SPI* in maize after insect feeding and SA induction. The results showed that SPI and key genes in the SA pathway exhibited a concurrent effect in transcription timing. After the transcription level of the SA signaling pathway transcription factor *NPR1* increased, the transcription level of *SPI* also increased. In contrast, the upregulation of *NPR3/4*, as inhibitory genes in the SA pathway, coincided with the termination of *SPI* upregulation. This further confirms that maize *SPI*, as a downstream defense gene of SA, is regulated by the SA pathway (Fig. 6).

### The source of the difference in growth inhibition of the *O. furnacalis* by different inbred lines of SPI

In our study, we evaluated the induction of resistance to *O. furnacalis* in different inbred lines of maize after SA induction and found significant differences in the induction of resistance among them (Fig. 2, Table 1). Moreover, the response speed and intensity of SA synthesis pathway genes were also different (Fig. 3). Based on Fig. 5, we found that the induction intensity of SPI in W22 maize after *O. furnacalis* feeding and SA induction was significantly higher than that in B73 and Mo17. This was also observed in the feeding deterrence assay (Fig. 7), where ZmSerpin-W exhibited the strongest feeding deterrence ability against *O. furnacalis*.

To investigate whether differences in the SA pathway among different maize inbred lines lead to differences in their resistance to *O. furnacalis*, we performed real-time fluorescent quantitative analysis of maize SA signaling pathway-related genes. Although the three maize inbred lines showed similar upregulation patterns, the upregulation levels of these genes differed among them. Specifically, *TGA1* in the W22 inbred line exhibited a significant difference compared to the other two inbred lines, showing an acceleration in transcription initiation speed after induction of resistance to *O. furnacalis*. *NPR3* in B73 also exhibited a significant difference in upregulation time compared to the other two inbred lines. The remaining genes mainly exhibited differences in upregulation levels. The differences in transcription levels and response rates of these genes may lead to differences in SPI expression levels, resulting in different resistance abilities among different maize inbred lines.

We performed a bioinformatics comparative analysis of *SPI* in different inbred lines of maize. The consistency of the *SPI* and protein sequences was low among the three inbred lines, indicating significant differences in *SPI* sequences among them. We also predicted the gene structure, conserved protein domains, and physicochemical properties of *SPI*. The results showed no significant differences in *SPI* structure and conserved domains among the three inbred lines, but significant differences were observed in their protein physicochemical properties and tertiary structures. This is similar to the results of Matthew *et al.* [40] study on the structure of the SPI superfamily protein, which showed that except for the highly conserved "center" of serpins, the superfamily has evolved into many new functional branches, explaining the differences in SPI protein structure and physicochemical properties among different maize inbred lines. This phenomenon is also observed in wheat, where the consistency of multiple Serpin gene sequences is low, but they have the same protein structure and function [41].

To further understand whether differences in *SPI* among different inbred lines affect protein activity, we cloned the ZmSerpin-B, ZmSerpin-M, and ZmSerpin-W genes in different inbred lines of maize. We constructed two expression vectors, pET-28a and pYES2, respectively. The protein expressed by pET-28a was unmodified and may affect protein activity [42, 43], whereas pYES2 can appropriately modify eukaryotic proteins to improve protein activity. We used both prokaryotic and eukaryotic expression vectors to construct the expression vectors. After inducing expression, we fed the *O. furnacalis* larvae with the bacterial suspension and found that the SPI protein expressed by the prokaryotic expression system significantly inhibited the growth and development of *O. furnacalis*, and there was no significant difference in the activity of SPI protein expressed by the pET-28a prokaryotic expression vector among different inbred lines (Fig. 7). After feeding the *O. furnacalis* larvae with the protein induced by the pYES2 eukaryotic system, significant differences were observed in the inhibition of *O. furnacalis* growth and development among different inbred lines. Therefore, we concluded that the differences in feeding deterrence activity of SPI among different inbred lines were not due to differences in *SPI* sequences and protein structures but in the main differences occur during the process of protein modification.

## Conclusion

In this experiment, we aimed to understand the reasons for the differences in insect resistance among different maize varieties. We screened and identified the insect resistance gene *SPI*, which is induced by both SA and *O. furnacalis* feeding stimuli. We conducted bioinformatics analysis, transcriptional level verification, and vector protein expression assays to elucidate the similarities and differences of SPI in different maize varieties. Additionally, we established the spatiotemporal regulatory relationship between the SA-responsive marker gene and *SPI* expression. The results showed that differences in SA signaling transcription factors, *SPI* transcription levels, and protein modifications of different SPIs are important factors influencing the variation in insect resistance among maize varieties. This study successfully identified and functionally validated the SA-induced SPI gene, providing valuable insights into plant responses to SA-induced insect resistance. The research may serve as a potential new strategy for controlling *O. furnacalis* invasion by enhancing the expression of the insect resistance gene *SPI* through exogenous SA induction.

## Materials and methods

### Plants and insects

Plant materials: In this study, nine maize inbred lines, including B104, B73, Chang7-2, Ep1, F7, Mo17, W22, Ye478, and Zheng58, were provided by the Institute of Maize Research, Heilongjiang Academy of Agricultural Sciences (Harbin, 150086, Heilongjiang, China).

Insect materials: The *O. furnacalis* used in this study was sourced from 1st instar larvae reared in our laboratory. The feeding method has been previously described in detail by Zhang *et al*. [44].

### Evaluation of insect resistance induced by SA in different maize inbred lines

The germinated maize seeds were individually planted in plastic containers measuring 15cm × 25cm, with a 2cm layer of soil on top, and grown outdoors. After the plants reached the three-leaf stage, they were subjected to different concentrations (0mM, 0.1mM, 0.5mM, and 1mM) and durations (2h, 6h and 9h) of SA foliage spray treatment. Maize leaves treated with different concentrations and durations of SA were cut and washed with distilled water, and then placed in plastic rearing boxes. Each box was infested with 50 first-instar *O. furnacalis* larvaes, and each treatment was replicated three times. The leaves in the rearing boxes were replaced every day, and the growth and development of the *O. furnacalis* and their adult lifespan were recorded. The *O. furnacalis* growth inhibition rate for each group was calculated every 5 days from the first day of the experiment until the pupal stage (Eq. 1). The optimal SA treatment conditions were selected based on the growth inhibition rate.

Based on the mortality rate of the *O. furnacalis*, a 0.5 mM SA solution was evenly sprayed onto the leaves of three-leaf-stage maize seedlings of different inbred lines. After 6 hours of treatment, the maize leaves were washed with distilled water, cut and placed in rearing boxes. Each box was infested with 50 second-instar *O. furnacalis* larvae, and each inbred line was replicated three times. Untreated maize leaves were used as the control group. The leaves in the rearing boxes were replaced every day, and the *O. furnacalis* larvae were weighed before and 5 days after treatment. The *O. furnacalis* growth inhibition rate for each group was calculated (Eq. 1).

1 $$Growth\;inhibition\;rate(\%)=\frac{Control\;weight\;gain-Treatment\;weight\;gain}{Treatment\;weight\;gain}\times100\%$$

Transcriptome data mining and screening of maize genes involved in insect resistance and feeding deterrence.

### Identification of maize *SPI* gene

Based on the transcriptome data of Longdan46 maize *O. furnacalis* feeding treatment, MeJA treatment and *O. furnacalis*-MeJA co-treatment (NCBI: PRJNA287429) [45], we found the upregulation of some genes only exclude MeJA treatment. We hypothesized that the upregulation of these genes might be regulated by the SA pathway, so we selected negative control (SRX1690323), OF feeding (SRX1690326), and JA-Of co-treatment (SRX1690325) data for comparison to identify potential insect resistance genes regulated by SA. Volcano plots and Venn diagrams were generated using the online tool Bioinformatics (http://www.bioinformatics.com.cn/), and heat maps were created using TBtools.

The genomic data of inbred maize lines were downloaded from the Maize Genomics Database (https://maizegdb.org/). Sequence alignment analysis of the SPI gene in different inbred maize varieties was performed using the DNAMAN software. Analysis of conserved motifs, domains, promoters, and introns and exons of the gene was carried out using TBtools software. Protein physical and chemical parameters were analyzed using EXPASY (https://web.expasy.org/protparam/). Prediction of protein signal peptides was performed using the online analysis tool SignalP-6.0 Server (https://services.healthtech.dtu.dk/services/SignalP-6.0/). Analysis of protein secondary structure was conducted using the online analysis tool PSIPRED (http://bioinf.cs.ucl.ac.uk/psipred/), and analysis of protein tertiary structure was performed using the online analysis tool PHYRE2 (http://www.sbg.bio.ic.ac.uk/phyre2/).

### RT-qPCR of *SPI* and SA pathway marker genes

We collected three-leaf stage maize seedlings and sprayed them with a 0.5 mM SA solution. After 6 hours treatment, we harvested the leaves. We then inoculated newly hatched maize larvae onto the maize seedlings, using insect clips for fixation. After 6 hours of feeding by the maize larvae, we collected the leaves. Untreated leaves were used as a control group. The collected leaves were rapidly frozen in liquid nitrogen and stored at -80°C. Total RNA was extracted from the leaves using the Trizol method, and the extracted RNA was reverse transcribed using the Prime Script^®^RT reagent kit (TaKaRa, Tokyo, Japan). Primers were designed and synthesized by TSINGKE Biological Technology (Harbin, China) (Tables S2, S3, and S4). TB Green^®^ Premix Ex Taq™ II (TliRNaseH Plus) reagent kit (TaKaRa, Tokyo, Japan) and QuantStudio™ 1 (Thermo Fisher Scientific, Waltham, MA, USA) instrument were used for fluorescence quantitative PCR. The reaction mixtures and programs for RT-qPCR are described in Table S5. The RT-qPCR data were analyzed using the 2^-ΔΔCt^ method to calculate the differences in expression levels of different maize *SPI* genes.

To investigate the transcriptional abundance of the *SPI* gene and SA conduction response marker genes in maize induced by SA, we first searched the SA signaling pathways in the KEGG online database (https://www.genome.jp/) and identified key genes. Then, we submitted these key genes to the maize genome database (https://maizegdb.org/) to obtain the target genes in the inbred lines. We downloaded the maize inbred line genome files from the maize genome database and used the TBTools tool to extract the CDS sequences of the target genes.

Next, we collected three-leaf stage maize seedlings from the B73, Mo17, and W22 lines. The leaves were harvested at 1, 2, 3, 6, 9, and 12 hours after SA induction (SA) and maize larval feeding (OF). The harvested leaves were stored in a refrigerator for further use. Total RNA was extracted from the leaves, and specific qPCR primers for the SA pathway marker genes were designed (Tables S2, S3, and S4). RT-qPCR was performed to analyze the gene expression levels.

### Construction of ZmSerpin expression vector in maize

Obtained the *SPI* gene sequences from the B73, Mo17, and W22 maize genome data, and named them *ZmSerpin-B*, *ZmSerpin-M*, and *ZmSerpin-W*, respectively. Full-length primers were designed using Primer5 software (Table S6), and *EcoR1* and *Not1* restriction enzyme sites were added to the 5' end of the upstream and downstream primers, respectively. Using maize cDNA as a template, we amplified the target genes with annealing temperature and extension time determined based on the primer's Tm value and GC content. The PCR reaction was performed using the Primer STAR^®^ Max DNA Polymerase kit (TaKaRa, Tokyo, Japan). Gel recovery and purification of the PCR products were carried out using the Tiangen Gel Purification Kit (column-based) (TIANGEN, Beijing, China). After gel purification, the PCR products and pET-28a vector were double-digested with *EcoR1* and *Not1* restriction enzymes. The digested target genes and pET-28a vector were then ligated using T4 DNA ligase. The recombinant plasmids containing the target genes were transformed into *E. coli* DH5α competent cells for amplification, and positive clones were selected using Kanamycin-containing culture medium. Plasmids from positive clones were extracted and subjected to PCR identification using T7-F (5'-TAATACGACTCACTATAGGG-3') and T7-R (5'-TGCTAGTTATTGCTCAGCGG-3') primers. The remaining bacterial culture was stored at 4°C. Plasmid extraction was performed using the TIANprep Mini Plasmid Kit (TIANGEN, Beijing, China). The recombinant plasmids were transformed into both *E. coli* BL21 and yeast expression vectors. Images were captured with the Tanon5200 Multi imaging system (Tanon, Shanghai, China).

### The induction expression and identification of the SPI protein

Successfully constructed *E. coli* single colony expressing the target protein was cultured in AMP-LB liquid medium at 37℃ with shaking at 220 rpm until reaching an OD_600_ =0.8. Then, IPTG was added to a final concentration of 1 mmol/L for induction, and the culture was further incubated at 25℃ with shaking at 120 rpm for 24 hours.

Identified yeast liquid (2 μL) was inoculated into SC-U+2% glucose liquid medium and cultured at 30℃ for 2 days. The culture was then centrifuged at 4000 rpm for 1 minute, and the sediment was resuspended in 10 mL of SC-U+2% lactose liquid medium to achieve an OD_600_ =0.4. This was followed by incubation at 30℃ for 3 days. After centrifugation at 4000 rpm for 1 minute, the sediment was resuspended in sterile water to achieve an OD_600_ =2.

The induced bacterial cells were collected and resuspended in 10 mL of 10 mM Tris (pH 8.0) buffer. The suspension was briefly placed in liquid nitrogen for 1 minute, slowly thawed at room temperature, subjected to repeated freeze-thaw cycles, and then lysed using sonication. After centrifugation at 4℃ and 12000 rpm for 15 minutes, the precipitate was collected for qualitative and quantitative analysis of the target protein characteristics using 15% SDS-PAGE polyacrylamide gel electrophoresis and protein molecular weight marker. Images were captured with the Tanon5200 Multi imaging system (Tanon, Shanghai, China), and protein bands were quantified using TanonImage software (Tanon, Shanghai, China). Afterwards, we adjust the concentration of the target protein to 0.2mg/ml.

### SPI protein bioactivity assay

Take 30g of artificial feed, add total mass 1mg of the target proteins expressed in *E. coli* BL21 (pET-28a-ZmSerpin-B, pET-28a-ZmSerpin-M, and pET-28a-ZmSerpin-W) and yeast (pYES2-ZmSerpin-B, pYES2-ZmSerpin-M, and pYES2-ZmSerpin-W), mix well, and let it stand under sterile conditions to maintain suitable moisture. The expression of proteins by pET-28a and pYES2 empty vectors served as positive controls, while water mixed with feed served as a negative control. The second instar *O. furnacalis* larvae were fed with the different treatments. Prior to the experiment, the larvae were starved for 12 hours. Each treatment included 50 larvae, and the experiment was repeated three times. The larvae were weighed before the experiment, and the feed was changed daily. After 5 days, the larvae were weighed again, and the growth inhibition rate of the *O. furnacalis* larvae was calculated (Eq. 1).

### Statistical analysis

All data were analyzed using GraphPad Prism version 9 and Origin 2021b. The results are presented as the means ± standard deviation (SD). Student's t-test was used to analyze the significant differential analysis of proteins expressed by the two expression vectors. One-way ANOVA followed Duncan’s test was used for comparison of growth inhibition rate among different treatments. All statistical analyses were conducted using SPSS software v20. *P-values* <0.05 were considered to be significant.

### Supplementary Information

**Additional file 1:** **Table S1.** Protein characteristics of maize SPI. **Table S2.** B73 maize inbred line Real-time PCR primers. **Table S3.** Mo17 maize inbred line Real-time PCR primers. **Table S4.** W22 maize inbred line Real-time PCR primers. **Table S5.** qPCR Reaction system and procedure. **Table S6.** Primer for SPI gene amplification in inbred lines. **Fig. S1.** Analysis of three different strains maize *SPI* genes and SPI proteins. **Fig. S2.** Cloning of *SPI* gene in *Zea Mays*. **Fig. S3.** Identification Results of *pET-28a-ZmSeRpin*and *pYES2-ZmSeRpin* vector digestion. **Fig. S4.** PCR of DH5α recombinant colonies. **Fig. S5.** PCR of BL21 and Yeast recombinant colonies. **Fig. S6.** SDS-PAGE electrophoresis of *E. coli* BL21 and yeast cells after induced expression.

## Data Availability

Data can be provided upon reasonable request from the corresponding author.
