# Supplementary material for: Salicylic acid inducing the expression of maize anti-insect gene SPI: a potential control strategy for Ostrinia furnacalis
Source: BMC Plant Biol. 2024 Feb 29;24:152. doi: 10.1186/s12870-024-04855-6 (PMC10902998; doi:10.1186/s12870-024-04855-6)
Supplement: Supplementary file 1 — Additional file 1: Table S1. Protein characteristics of maize SPI. Table S2. B73 maize inbred line Real-time PCR primers. Table S3. Mo17 maize inbred line Real-time PCR primers. Table S4. W22 maize inbred line Real-time PCR primers. Table S5. qPCR Reaction system and procedure. Table S6. Primer for SPI gene amplification in inbred lines. Fig. S1. Analysis of three different strains maize SPI genes and SPI proteins. Fig. S2. Cloning of SPI gene in Zea Mays. Fig. S3. Identification Results of pET-28a-ZmSeRpinand pYES2-ZmSeRpin vector digestion. Fig. S4. PCR of DH5α recombinant colonies. Fig. S5. PCR of BL21 and Yeast recombinant colonies. Fig. S6. SDS-PAGE electrophoresis of E. coli BL21 and yeast cells after induced expression. [file 12870_2024_4855_MOESM1_ESM.docx]

**Supporting Information**

**Table S1.** Protein characteristics of maize SPI

| Protein name | Amino acids (aa) | molecular weight  (KDa) | PI | GRAVY | Instability index | signal peptide（%） |
| --- | --- | --- | --- | --- | --- | --- |
| B73 SPI | 360 | 38.81 | 5.79 | 0.046 | 38.34 | 0.15 |
| Mo17 SPI | 361 | 39.10 | 10.01 | -0.210 | 46.52 | 0.00 |
| W22 SPI | 397 | 42.15 | 5.52 | 0.076 | 35.84 | 0.06 |

**Table S2.** B73 maize inbred line Real-time PCR primers

| Primers | Sequences (5'-3') | TM (℃) | Product (bp) |
| --- | --- | --- | --- |
| *SPI-*F | AAGAGATTGGTGCTCTGCCC | 60.03 | 415 |
| *SPI-*R | CGCGAAATTTCCGTCCCATC | 59.97 |  |
| *NPR1-*F | AAGTTGCTTCCCACTCAGGG | 59.89 | 184 |
| *NPR1-*R | TGCACAGGGTCCGAAATGAA | 59.89 |  |
| *NPR3-*F | GGCGAAGACAGACTTCCACT | 59.68 | 513 |
| *NPR3-*R | ATTTTGGACCACGCTTGCAC | 59.97 |  |
| *NPR4-*F | TACATGCAGACGACGGTACG | 59.9 | 289 |
| *NPR4-*R | ACGTTGCATTTGCCAGTGTC | 59.97 |  |
| *TGA1-*F | CATCCAAGACTGGAGGCGAG | 60.18 | 307 |
| *TGA1-*R | AGAACATCTTACTGCCGCCG | 60.46 |  |
| *CYP71-*F | CTTCTGAAGGCCATCCTCGG | 60.18 | 713 |
| *CYP71-*R | AACCGGTCGAACATCTTGCT | 59.97 |  |
| *ICS1-*F | GCATCATCCGCATCGAGGT | 60.3 | 243 |
| *ICS1*-R | CGCGCCAATCAGCTAGAGAA | 60.53 |  |
| *MDL1*-F | GGTAGGAGAGGGTCGTCTGT | 60.03 | 174 |
| *MDL1-*R | CCGAAAACTCCCATCGACCA | 60.04 |  |
| *PAL-*F | GTCGTCCACCTACATCGTGG | 60.18 | 96 |
| *PAL-*R | ACCTGGGTCACGGTGTTCTT | 61.34 |  |
| *WRKY28-*F | TACCAATGGCGGAAGTACGG | 59.82 | 297 |
| *WRKY28-*R | TTGTTTCTGCTGGTGCTGGT | 60.4 |  |
| *WRKY46-*F | GGTGTACGACGTGATCTACCA | 59.26 | 159 |
| *WRKY46-*R | CCCTCCTCCGTTTCCGTCTT | 61.54 |  |
| *SIPK-*F | TGCTGCCATTGATGTCTGGT | 59.96 | 71 |
| *SIPK-*R | CAGGAAACAGGGGTTGACGA | 59.89 |  |

**Table S3.** Mo17 maize inbred line Real-time PCR primers

| Primers | Sequences (5'-3') | TM (℃) | Product (bp) |
| --- | --- | --- | --- |
| *SPI-*F | AAGAGATTGGTGCTCTGCCC | 60.03 | 415 |
| *SPI-*R | CGCGAAATTTCCGTCCCATC | 59.97 |  |
| *NPR1-*F | CCCTGAGCATGACAAGAGGG | 60.11 | 151 |
| *NPR1-*R | CACCTTGGGCTCACAGTAGG | 60.04 |  |
| *NPR3-*F | TGGGAAAGCCGATCCAACTC | 60.04 | 251 |
| *NPR3-*R | GCTTTAGCGGATCTTGCGTG | 59.97 |  |
| *NPR4-*F | CCAACACGTCCTTAGTGGCT | 59.97 | 304 |
| *NPR4-*R | TACAAACCAGTCGCAAACGC | 59.69 |  |
| *TGA1-*F | CATCCAAGACTGGAGGCGAG | 60.18 | 307 |
| *TGA1-*R | AGAACATCTTACTGCCGCCG | 60.46 |  |
| *CYP71-*F | CTTCTGAAGGCCATCCTCGG | 60.18 | 713 |
| *CYP71-*R | AACCGGTCGAACATCTTGCT | 59.97 |  |
| *ICS1-*F | GCATCATCCGCATCGAGGT | 60.3 | 243 |
| *ICS1-*R | CGCGCCAATCAGCTAGAGAA | 60.53 |  |
| *MDL1-*F | GGTAGGAGAGGGTCGTCTGT | 60.03 | 174 |
| *MDL1*-R | CCGAAAACTCCCATCGACCA | 60.04 |  |
| *PAL-*F | GTCGTCCACCTACATCGTGG | 60.18 | 96 |
| *PAL-*R | ACCTGGGTCACGGTGTTCTT | 61.34 |  |
| *WRKY28-*F | TTGGCCGTCAGGACGAAAG | 60.3 | 473 |
| *WRKY28-*R | CCGTACTTCCGCCATTGGTA | 59.82 |  |
| *WRKY46-*F | GGTGTACGACGTGATCTACCA | 59.26 | 159 |
| *WRKY46-*R | CCCTCCTCCGTTTCCGTCTT | 61.54 |  |
| *SIPK-*F | CTGCTGCCATTGATGTCTGG | 59.26 | 88 |
| *SIPK-*R | TGGATGTAATCCCGTCCAGG | 58.87 |  |

**Table S4.** W22 maize inbred line Real-time PCR primers

| Primers | Sequences (5'-3') | TM (℃) | Product (bp) |
| --- | --- | --- | --- |
| *SPI-*F | AAGAGATTGGTGCTCTGCCC | 60.03 | 415 |
| *SPI-*R | CGCGAAATTTCCGTCCCATC | 59.97 |  |
| *NPR1-*F | AAGTTGCTTCCCACTCAGGG | 59.89 | 184 |
| *NPR1-*R | TGCACAGGGTCCGAAATGAA | 59.89 |  |
| *NPR3-*F | AGGTTGGGAAAGTCGATCCAA | 59.3 | 165 |
| *NPR3-*R | CGCCCATCATCGACATCTCA | 59.97 |  |
| *NPR4-*F | CCAACACGTCCTTAGTGGCT | 59.97 | 304 |
| *NPR4-*R | TACAAACCAGTCGCAAACGC | 59.69 |  |
| *TGA1-*F | CATCCAAGACTGGAGGCGAG | 60.18 | 307 |
| *TGA1-*R | AGAACATCTTACTGCCGCCG | 60.46 |  |
| *CYP71-*F | CTTCTGAAGGCCATCCTCGG | 60.18 | 713 |
| *CYP71-*R | AACCGGTCGAACATCTTGCT | 59.97 |  |
| *ICS1-*F | GAGTGGTTGCACACGCAGA | 60.89 | 186 |
| *ICS1-*R | AGCGAGAGAAAAGGGTTCCG | 60.04 |  |
| *MDL1-*F | GGTAGGAGAGGGTCGTCTGT | 60.03 | 174 |
| *MDL1-*R | CCGAAAACTCCCATCGACCA | 60.04 |  |
| *PAL-*F | GTCGTCCACCTACATCGTGG | 60.18 | 96 |
| *PAL-*R | ACCTGGGTCACGGTGTTCTT | 61.34 |  |
| *WRKY28-*F | TACCAATGGCGGAAGTACGG | 59.82 | 297 |
| *WRKY28-*R | TTGTTTCTGCTGGTGCTGGT | 60.4 |  |
| *WRKY46-*F | CCGCGCCCTTGTACCTC | 60.18 | 268 |
| *WRKY46-*R | AAGCTGGCGATGTCGTCTAT | 59.25 |  |
| *SIPK-*F | CTGCTGCCATTGATGTCTGG | 59.26 | 88 |
| *SIPK-*R | TGGATGTAATCCCGTCCAGG | 58.87 |  |

**Table S5.** qPCR Reaction system and procedure

| Reagent | | Volume(μL) |
| --- | --- | --- |
| TB Green Premix Ex Taq II（Tli RNaseH Plus）（2X） | | 10 |
| PCR Forward Primer（10 μM） | | 0.8 |
| PCR Reverse Primer（10 μM） | | 0.8 |
| ROX Reference Dye（50×） | | 0.4 |
| cDNA | | 2 |
| RNase free ddH_2_O | | 6 |
| Total | | 20 |
| Steps | Cycles | Procedure |
| hold Stage | 1 | 95℃ 3 min |
| PCR Stage | 40 | 95℃ 15 s |
|  |  | 60℃ 1 min |
| Melt Curve Stage | 1 | 95℃ 15 s |
|  |  | 60℃ 1 min |
|  |  | 95℃ 1 s |

**Table S6. Primer for *SPI* gene amplification in inbred lines**

| Primers | Sequences (5’-3’) | Tm (℃) | Product (bp) |
| --- | --- | --- | --- |
| *ZmSerpin-B*-F | CGGAATTCATGTCATCATGGCATCAAGA | 53.7 | 1083 |
| *ZmSerpin-B*-R | AAAAGGAAAGCGGCCGCTTAGGAATGAAGCCGGTCT | 54.7 |  |
| *ZmSerpin-M*-F | CCGGAATTCATGTCATCATGGCATCAAGACG | 53.7 | 1086 |
| *ZmSerpin-M*-R | AAAAGGAAAGCGGCCGCTCACTCCGCTAGT | 52 |  |
| *ZmSerpin-W*-F | CCGGAATTCATGTCATCATGGCATCAAGA | 60.6 | 1194 |
| *ZmSerpin-W*-R | AAAAGGAAAGCGGCCGCTCACTCCGCTAG | 56.2 |  |





**Fig. S1** Analysis of three different strains maize *SPI* genes and SPI proteins

A: Analysis of *SPI* structure in different maize strains (a: Gene conserved Motif, b: Conserved domains of genes, c: Gene promoter analysis, d: Gene CDS and UTR analysis). B: Alignment of *SPI* gene sequences of maize inbred lines. C: Secondary structural models of SPI proteins in three maize inbred lines. D: Tertiary structural model of SPI protein in three maize inbred line. E: Alignment of SPI protein sequences of three maize inbred lines.


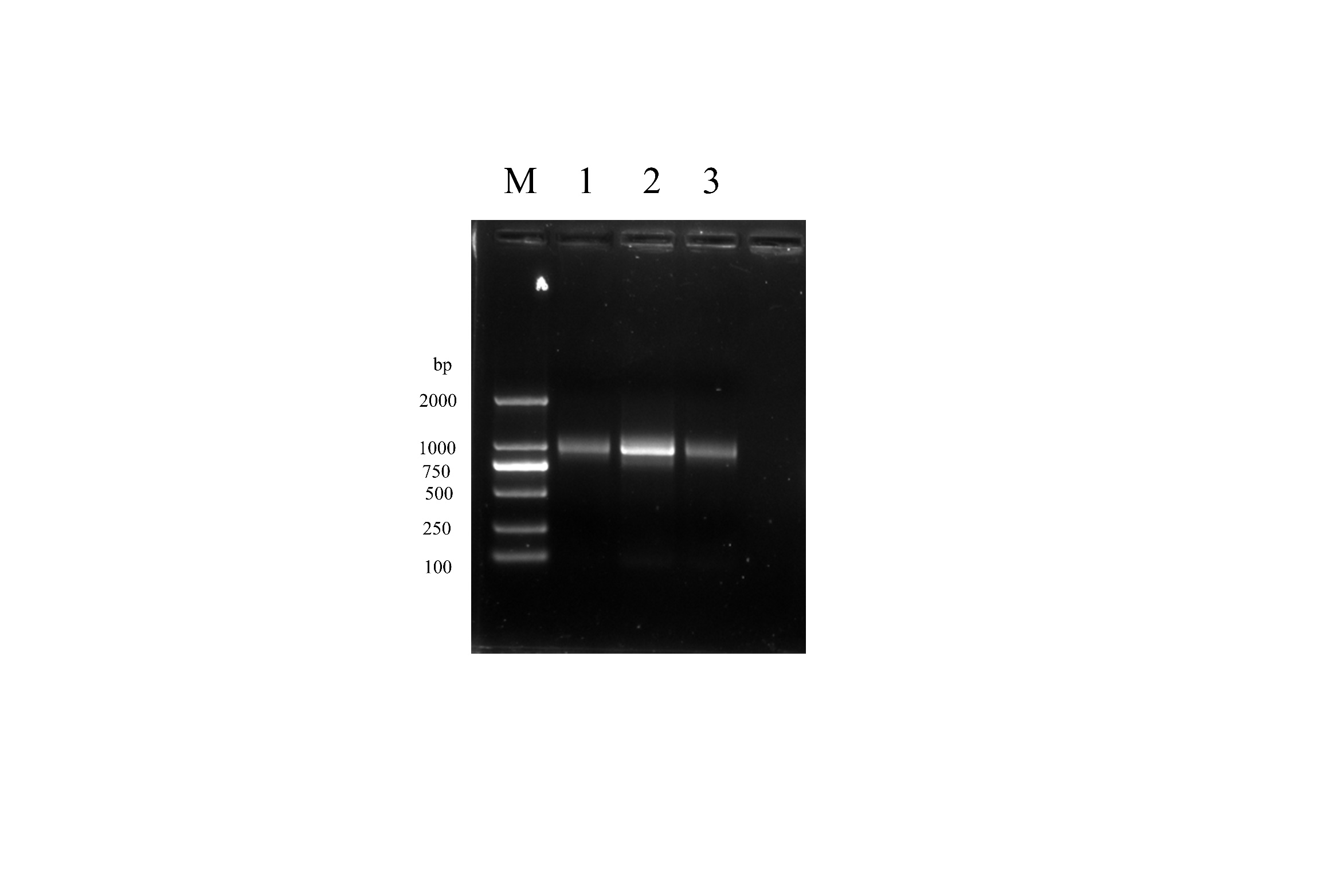


**Fig. S2** Cloning of *SPI* gene in *Zea Mays*

M: DL2000 marker; 1: *ZmSeRpin-B*; 2: *ZmSeRpin-M*; 3: *ZmSeRpin-W*


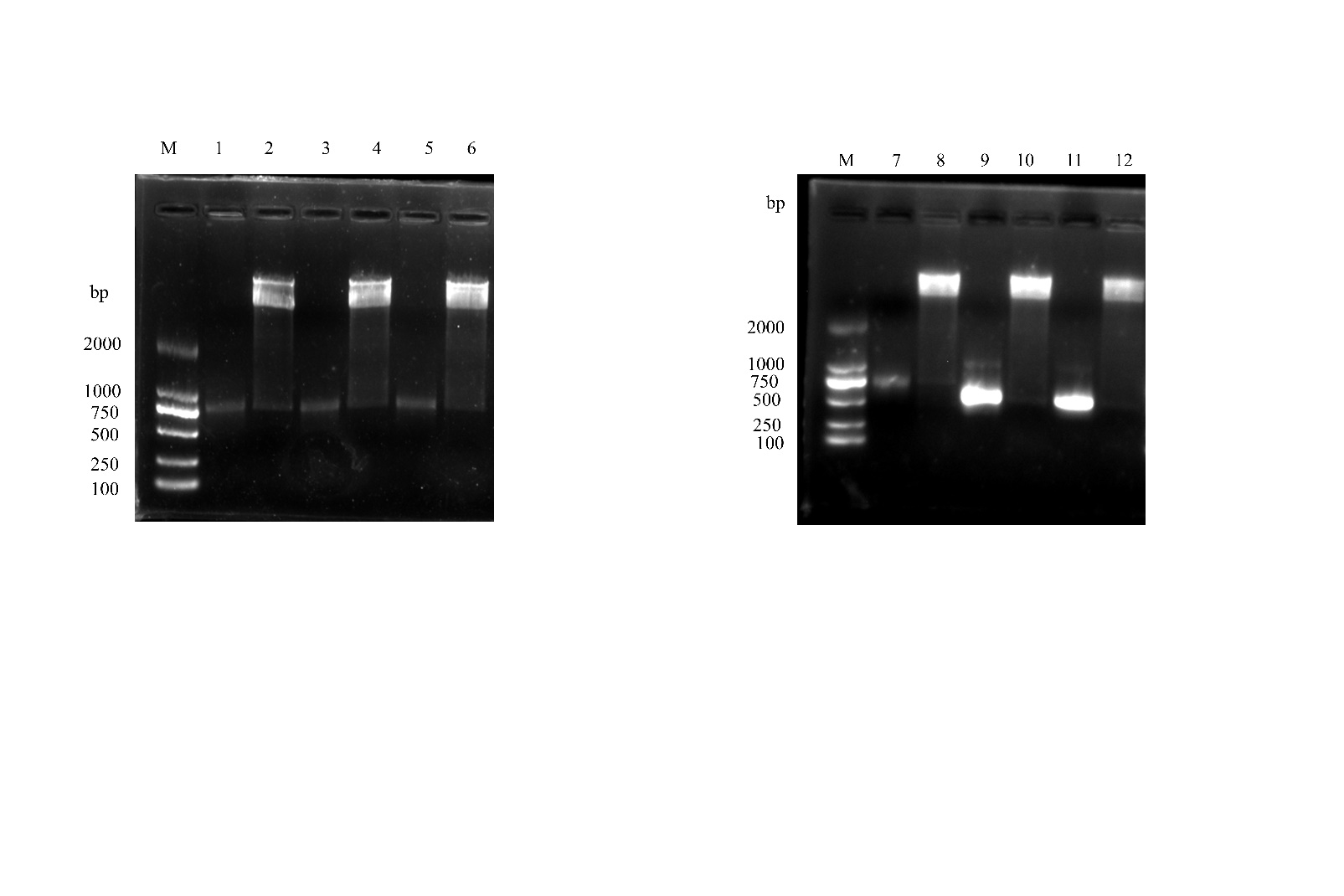


**Fig. S3** Identification Results of *pET-28a-ZmSeRpin* and *pYES2-ZmSeRpin* vector digestion

M: DL2000 marker; 1: *ZmSeRpin-B*; 2: *pET-28a-ZmSeRpin-B*; 3: *ZmSeRpin-M*; 4: *pET-28a-ZmSeRpin-M*; 5: *ZmSeRpin-W*; 6: *pET-28a-ZmSeRpin-W*; 7: *ZmSeRpin-B*; 8: *pYES2-ZmSeRpin-B*; 9: *ZmSeRpin-M*; 10: *pYES2-ZmSeRpin-M*; 11: *ZmSeRpin-W*; 12: *pYES2-ZmSeRpin-W.*


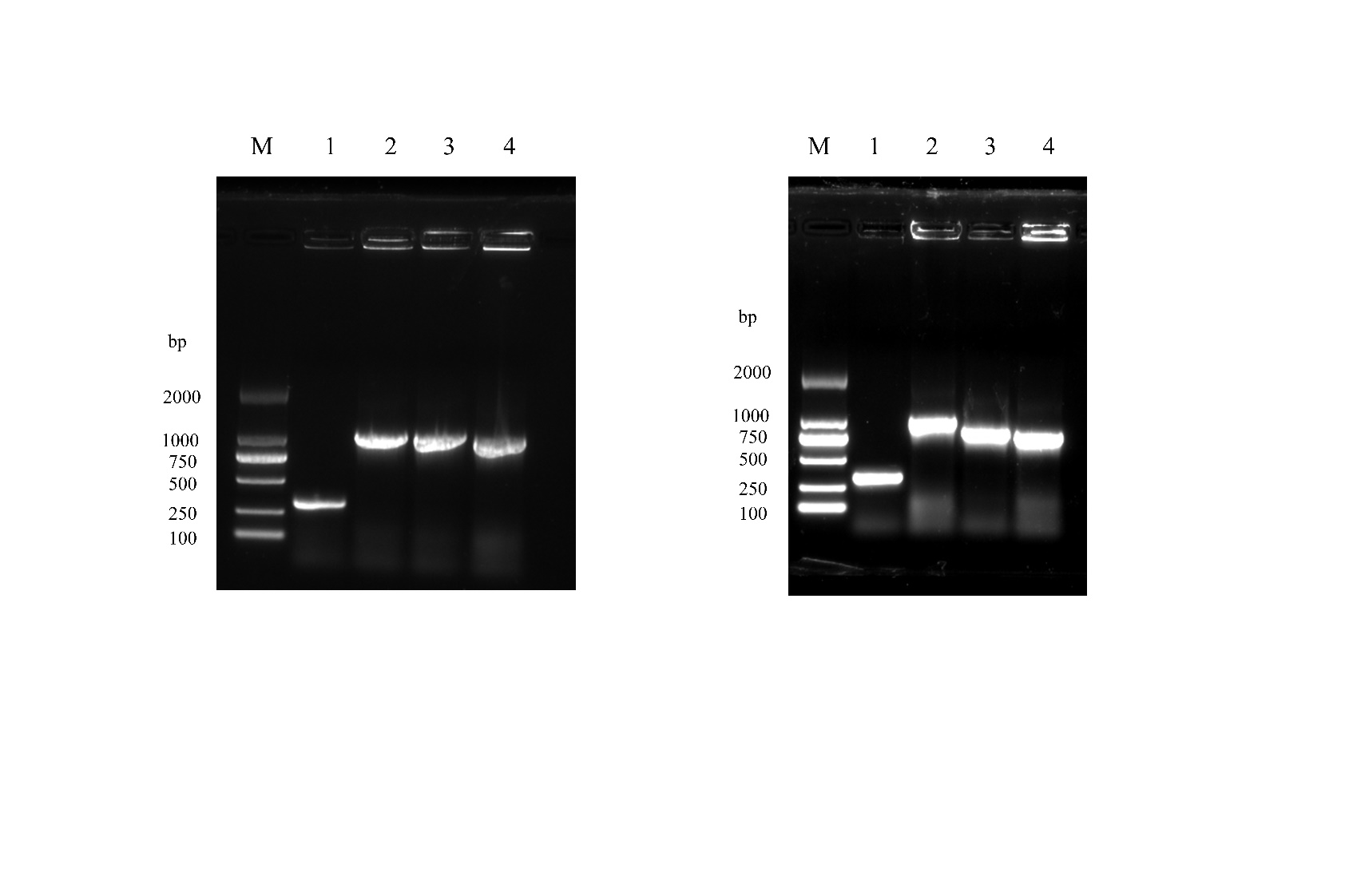


**Fig. S4** PCR of DH5α recombinant colonies

M: DL2000 Marker; 1: pET-28a; 2: *pET-28a-ZmSeRpin-B*; 3: *pET-28a-ZmSeRpin-M*; 4: *pET-28a-ZmSeRpin-W*; 5: pYES2; 6: *pYES2-ZmSeRpin-B*; 7: *pYES2-ZmSeRpin-M*; 8: *pYES2-ZmSeRpin-W.*


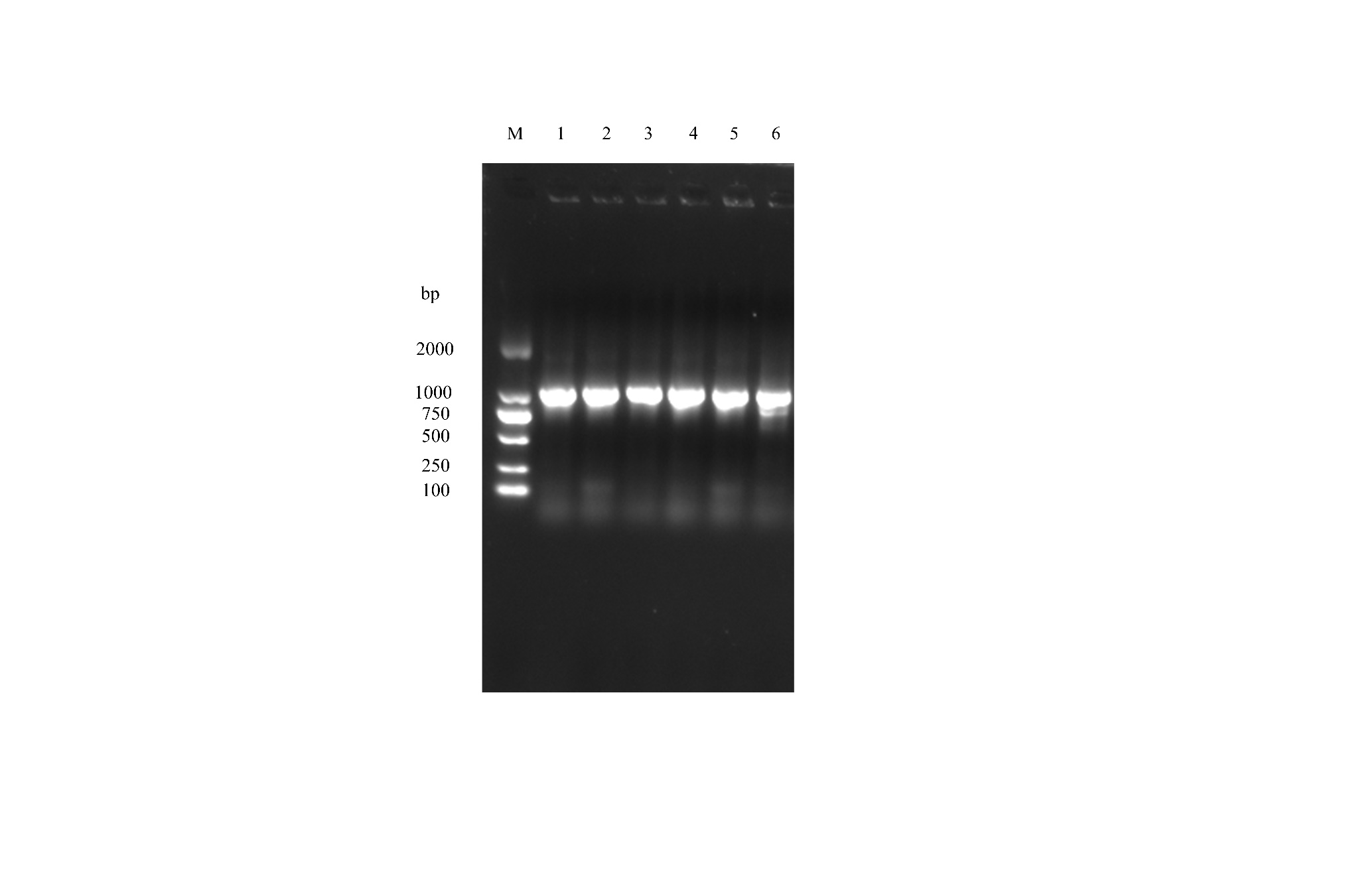


**Fig. S5** PCR of BL21 and Yeast recombinant colonies

M: DL2000 Marker; 1: *pET-28a-ZmSeRpin-B*; 2: *pET-28a-ZmSeRpin-M*; 3: *pET-28a-ZmSeRpin-W*; 4: *pYES2-ZmSeRpin-B*; 5: *pYES2-ZmSeRpin-M*; 6: *pYES2-ZmSeRpin-W.*


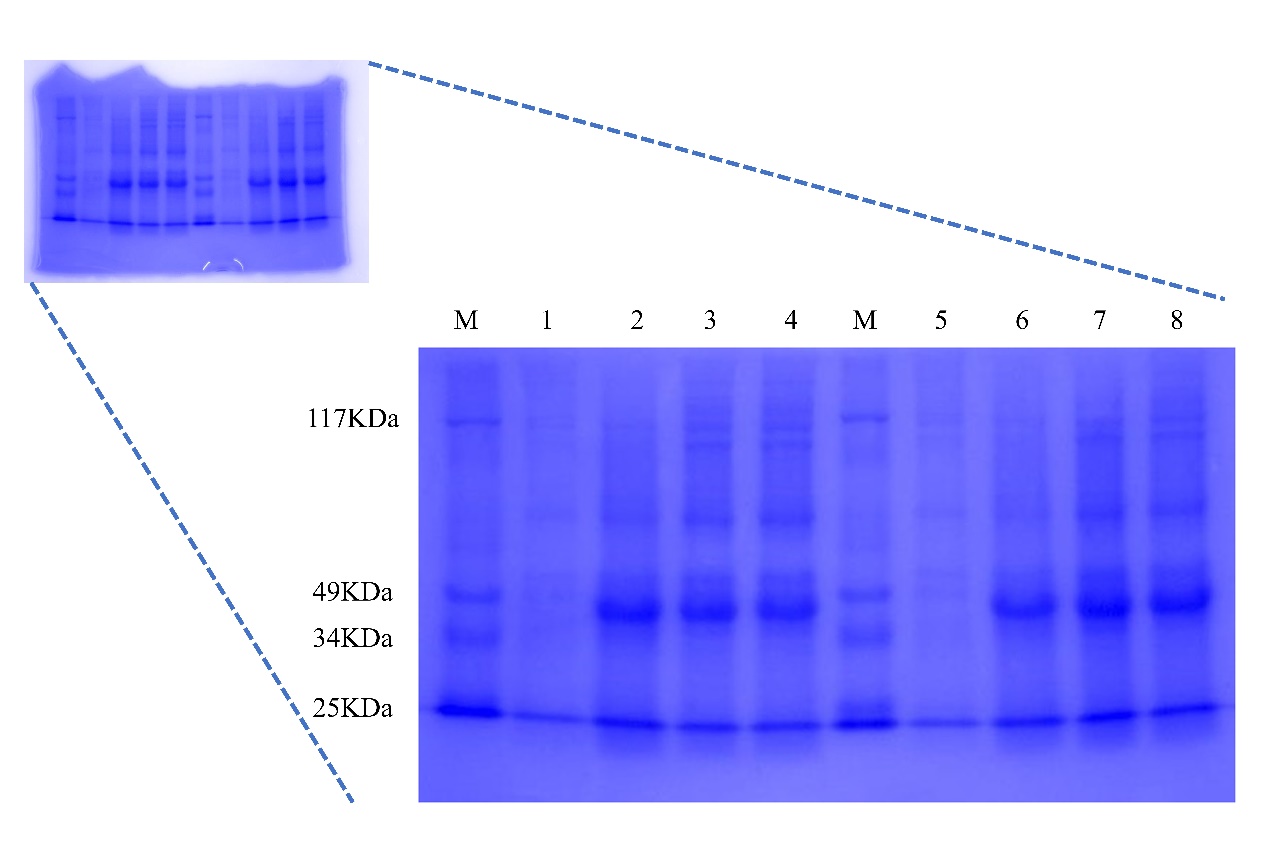


**Fig. S6** SDS-PAGE electrophoresis of *E. coli* BL21 and yeast cells after induced expression

M: 117KDa marker; 1: pET-28a; 2: pET-28a-ZmSeRpin-B; 3: pET-28a-ZmSeRpin-M; 4: pET-28a-ZmSeRpin-W; 5: pYES2; 6: pYES2-ZmSeRpin-B; 7: pYES2-ZmSeRpin-M; 8: pYES2-ZmSeRpin-W.
